# Combining multiple hypothesis testing and affinity propagation clustering leads to accurate, robust and sample size independent classification on gene expression data

**DOI:** 10.1186/1471-2105-13-270

**Published:** 2012-10-17

**Authors:** Argiris Sakellariou, Despina Sanoudou, George Spyrou

**Affiliations:** 1 Biomedical Informatics Unit, Biomedical Research Foundation of the Academy of Athens, Athens, Greece; 2 Department of Informatics and Telecommunications, National & Kapodistrian Univ. of Athens, Athens, Greece; 3 Pharmacology Department, Medical School, National & Kapodistrian Univ. of Athens, Athens, Greece

## Abstract

**Background:**

A feature selection method in microarray gene expression data should be independent of platform, disease and dataset size. Our hypothesis is that among the statistically significant ranked genes in a gene list, there should be clusters of genes that share similar biological functions related to the investigated disease. Thus, instead of keeping *N* top ranked genes, it would be more appropriate to define and keep a number of gene cluster exemplars.

**Results:**

We propose a hybrid FS method (m*AP*-KL), which combines multiple hypothesis testing and affinity propagation (AP)-clustering algorithm along with the Krzanowski & Lai cluster quality index, to select a small yet informative subset of genes. We applied m*AP*-KL on real microarray data, as well as on simulated data, and compared its performance against 13 other feature selection approaches. Across a variety of diseases and number of samples, m*AP*-KL presents competitive classification results, particularly in neuromuscular diseases, where its overall AUC score was 0.91. Furthermore, m*AP*-KL generates concise yet biologically relevant and informative *N*-gene expression signatures, which can serve as a valuable tool for diagnostic and prognostic purposes, as well as a source of potential disease biomarkers in a broad range of diseases.

**Conclusions:**

m*AP*-KL is a data-driven and classifier-independent hybrid feature selection method, which applies to any disease classification problem based on microarray data, regardless of the available samples. Combining multiple hypothesis testing and AP leads to subsets of genes, which classify unknown samples from both, small and large patient cohorts with high accuracy.

## Background

Microarray data analysis is widely used for the identification of ‘informative’ genes. However, due to the ‘curse’ of dimensionality, where the number of gene probes represented on microarrays far exceeds the available number of cases (samples) as well as the inherent noise in microarray data, feature selection (FS) approaches strive to achieve this goal. Typically, informative genes are selected according to a two-sample statistical test combined with multiple testing procedures to guard against Type 1 errors [1]. This methodology generates gene lists, which then can be either ranked or filtered according to certain statistical criteria, e.g. p-value, q-value etc. The selected subset of genes is assumed to construct better classifiers, both in terms of accuracy and efficiency. In particular, we expect improved classification performance and generalization by avoiding over-fitting. Furthermore, the classifiers will be more efficient in time and space because of the fewer features, and biologists’ insights will be augmented [2].

A wide variety of FS algorithms has been proposed [3-5] and depending on how they combine the feature selection search with the construction of the classification model they can be classified into 3 categories: filter, wrapper, and embedded [2]. A filter based algorithm either selects features through univariate rankings [6-8] or incorporates feature dependencies (multivariate) like Correlation based Feature Selection (CFS) [9]. On the other hand, wrapper algorithms like genetic algorithms [10-12], attempt to select multiple features simultaneously based on their performance on a training set [13]. Finally, embedded algorithms, like Random Forest [14], select the best subset of genes incorporating the classifiers’ bias [2].

Complementary to this categorization, hybrid approaches have drawn researchers’ interest. Specifically the benefits of usually two different techniques are combined towards the identification of an improved gene subset selection, for example, a univariate filter with a wrapper or an embedded method [15-20]. Apart from FS methods, there are also data reduction techniques such as principal component analysis and partial least squares, which search for linear combinations of all genes to provide us with a small subset of ‘metagenes’ [21].

An FS algorithm should perform efficiently and independently of the sample size and yield its subset within a reasonable period, to enable numerous experiments. Moreover, the subset’s length should be small, for instance, less than 50 genes, and the selected genes should present biological relevance to the inspected disease so as to facilitate further analysis. Despite the plethora of available FS methods, none of them has managed to successfully deal with all the aforementioned issues playing the role of a milestone. For instance, some methods are effective with small cohorts while others with large ones [22]. Aside from this, there are methods that are developed and tested for specific diseases, leaving their suitability for broader use unexplored [23]. Furthermore, some FS algorithms are so sophisticated that they either need specialized and expensive hardware to operate or an impractically long run time [12].

We propose a data-driven and classifier-independent hybrid FS method (m*AP*-KL), which combines multiple hypothesis testing [24] and affinity propagation (AP) clustering algorithm [25] in conjunction with the Krzanowski & Lai [26] cluster quality index, to select a small subset of informative genes. Our hypothesis is that among the statistically significant genes there should be clusters of genes that share similar biological functions related to the investigated disease. Thus, instead of keeping a number of the top ranked genes, it would be more appropriate to define and keep a number of gene cluster exemplars. We tested m*AP*-KL on real data from small and large cohorts, as well as on simulated data, and compared its performance against 13 other FS approaches. According to the results, m*AP*-KL achieves competitive classification results, particularly in the neuromuscular disease data, as well as in breast and colon cancers data, with subsets of less than 20 genes in most of the cases.

## Methods

### Rationale for selecting the proposed approach

Jaeger et al. [16] claimed that ranking algorithms produce lists of genes, where the top ranked genes are highly correlated with each other, mainly because they belong to the same pathway. Additionally, Hall in his thesis [9] investigated the hypothesis that “A good feature subset is one that contains features highly correlated with the class, yet uncorrelated with each other”.

So far several approaches have been proposed [16,27-29] based on these beliefs with promising classification results [28,29]. However, certain methodological differences or limitations prompt the development of our approach. The order of the analysis steps (ranking and clustering), the number of informative genes, and the data manipulation are issues that need specific focus in such an analysis. Our method uses ranking prior to clustering, similarly to HykGene [28] and mRMR [29] and contrary to Jaeger and Hanczar [27], because we wanted to filter the statistically redundant genes to facilitate the clustering analysis. Regarding the number of genes, we employ a clustering index to determine the ‘actual’ number of representative genes. This differs from mRMR method, which iterates in its ranked gene list before concluding to a subset, and from Jaeger and Hanczar, where the resultant subset is driven by the initial number of potential clusters, which is set arbitrarily. In relation to HykGene, we determine the number of clusters and thus the ‘representative genes’ irrespectively of the classifier employed. Finally, apart from the necessary transformation and normalization on the raw intensity values we do not perform any further preprocessing manipulation, like discretization as mRMR does to improve the classification results.

### The general framework and implementation of our methodology

The proposed methodology combines ranking-filtering and cluster analysis to select a small set of non-redundant but still highly discriminative genes. In relation to the filtering step, we first employ the maxT function (see Feature selection methods) from the ‘multtest’ package to rank the genes of the training set and then we reserve the top *N* genes (*N* = 200) for further exploitation. We based our decision on keeping only the top 200 genes on the findings of a previous study [30], where we observed a weak impact on the classification performance when differentiating the subset’s length.

In turn, prior to clustering analysis with AP we have to define the number of clusters, which in essence will be the number of representative genes that finally will compose our subset. We apply the index of Krzanowski and Lai as included in the ‘ClusterSim’ package [31] to determine the number of clusters solely on the disease samples of the training test set. Krzanowski and Lai is defined by

(1) $$DIFF\left(k\right)={\left(k-1\right)}^{2/p}W_{k-1}-k^{2/p}W_{k'}$$

when choosing the number of clusters (k) to maximize the quantity $KL\left(k\right)=\left|\frac{DIFF\left(k\right)}{DIFF\left(k+1\right)}\right|\text{.}$ The W_k_ denotes the within-cluster sum of squared errors.

The final step of our methodology involves the cluster analysis. For this task, we engage the AP clustering method, which detects *n* (*n* = *k*, the Krzanowski and Lai index) clusters among the top *N* genes, according to the pre-defined number, and provides us with a list of the most representative genes of each cluster, the so called exemplars. These *n* exemplars are expected to form a classifier that shall discriminate between the normal and disease classes in a test set. Finally, we formulate the test set by keeping only those *n* genes, and proceed with the classification.

**Figure 1:**
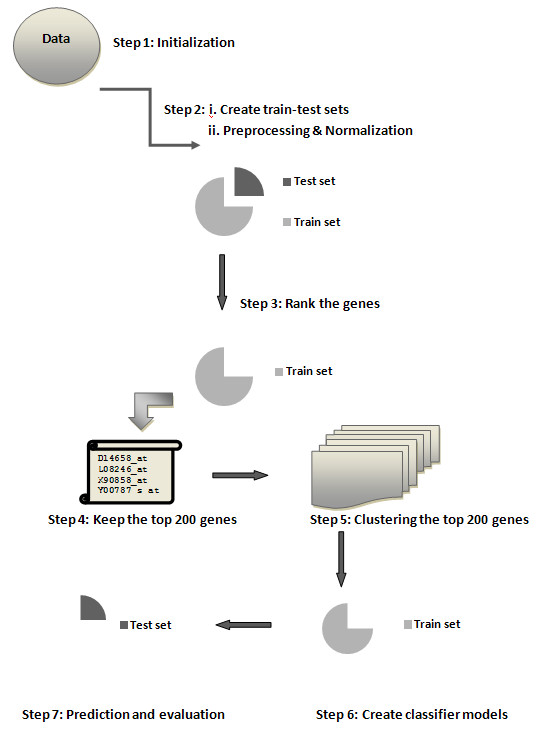
**The m*AP*-KL methodology flowchart** .

The m*AP*-KL is developed under the R environment [32], in which we first incorporated the ‘multtest’, ‘ClusterSim’, and ‘APCluster’ [33] packages, and then created a function (see Supplementary) to implement our methodology. The general flowchart of our methodology appears in Figure 1.

### Affinity propagation clustering method

Affinity propagation identifies a set of centers (exemplars) from actual data points. Contrary to k-centers technique, which begins with an initial set of randomly selected exemplars and iteratively refines this set to decrease the sum of squared errors, AP considers each data point as a node in a network, and recursively transmits real-valued messages along edges of the network until a good set of exemplars and corresponding clusters emerges. At any point in time, the magnitude of each message reflects the current affinity that one data point has for choosing another data point as its exemplar.

Messages exchanged between data points can be of two kinds: ‘responsibility’ r(i,k), and ‘availability’ a(i,k). ‘Responsibility’ reflects the accumulated evidence for how well-suited point k is to serve as the exemplar for point i, taking into account other potential exemplars for point i. On the other hand, ‘availability’ reflects the accumulated evidence for how appropriate it would be for point i to choose point k as its exemplar, taking into account the support from other points that point k should be an exemplar. Initially, the availabilities are set to zero. AP can be applied to problems where the similarities are neither symmetric nor satisfy the triangle inequality [25].

### Classification and evaluation

Regarding the classification phase we employed SVM [34] with linear kernel, KNN [35], and RF [14] classifiers under the WEKA [36] environment, to evaluate the performance of all FS methods employed. We first conducted a 5-fold cross-validation (5-CV) on the training sets to assess the potential classification strength of the models’ and then estimated its prediction power on the separate test sets.

To evaluate the classification results, we employed various standard performance measures, which provide different insights. Accuracy (ACC) is one of the most popular performance measures in machine learning classification, though it does not take into account the nature of the incorrect predictions, which can be crucial in clinical medicine and totally misleading about the actual classification performance of a given classifier. Therefore we engaged the area under the receiver operating characteristics (ROC) curve or in short AUC, which has been introduced as a better measure for evaluating the predictive ability of machine learners than accuracy [37]. The ROC curve is a two-dimensional plot between the TPR (Y-axis) against the FPR (X-axis) of the predictions. The closer the curve is to the Y-axis (high true positives) and the further away it is from the X-axis (low false positives), the more accurate the predictions are [38].

Additionally, we employed true negative rate (TNR) or specificity*,* which represents the ratio of correctly classified negatives to the actual number of negatives and controls type I errors, as well as true positive rate (TPR) or sensitivity, which is defined to be the ratio of positives correctly classified to the actual number of positives and controls type II errors. Both, specificity and sensitivity are mutually independent [39]. The combination of those three measures provides us with an adequate overview of the classification’s performance.

### Datasets illustration

#### Microarray data

In this study, we utilized real and synthetic data to assess m*AP*-KL’s performance. Neuromuscular and cancer diseases data comprise the real microarray data and are available in comma-delimited format in the Supplementary section. Neuromuscular diseases are rare among the general population, thus the available tissue samples and whole transcriptome data are very limited. This characteristic is crucial since we intended to develop a FS method that produces robust models even in studies with limited number of samples. We therefore included data from Bakay et al. [40] related to ‘amyotrophic lateral sclerosis’ (ALS), ‘Duchenne muscular dystrophy’ (DMD), ‘juvenile dermatomyositis’ (JDM), ‘limb-girdle muscular dystrophy type 2A’ (LGMD2A), and ‘limb-girdle muscular dystrophy type 2B’ (LGMD2B), as well as ‘nemaline myopathy’ (NM) data from Sanoudou and Beggs [41] and Sanoudou et al. [42]. The gene expression data for the first five diseases originate from Affymetrix HG_U133A gene chips and share a set of 18 normal samples, whereas the NM data originate from Affymetrix HG_U95A gene chips and have been compared to 21 normal samples. We divided the data approximately in half, and kept the first half to build a balanced train sets and the second half to validate the classification models (Table 1). Concerning the pre-processing approach, all neuromuscular data underwent log2 transformation and quantile normalization across samples.

**Table 1:** The real microarray data divided in train and test sets

**Datasets**	**Attributes (nr of genes)**	**Train set samples (class1:class2)**	**Test set samples (class1:class2)**
Amyotrophic lateral sclerosis **(als)**	22,283	6:6	12:3
Duchenne muscular dystrophy **(dmd)**	22,283	7:7	11:3
Juvenile dermatomyositis **(jdm)**	22,283	10:10	8:11
Limb-girdle muscular dystrophy type 2A **(lgmd2a)**	22,283	7:7	11:3
Limb-girdle muscular dystrophy type 2B **(lgmd2b)**	22,283	7:7	11:3
Nemaline myopathy **(nm)**	12,600	8:8	13:5
**breast cancer**	(4348)24,481	44:34	7:12
**colon cancer**	7,129	15:15	7:25
**all/aml leukemia**	7,129	27:11	20:14
**prostate cancer**	*12,600*	52:50	25:9

Regarding the cancers datasets, we utilized microarray data from breast cancer, colon cancer, leukemia, and prostate cancer, all of which are considered benchmark datasets and have been widely used in gene expression-classification studies. Van’t Veer [23] explored breast cancer patients’ clinical outcome following modified radical mastectomy or breast-conserving treatment combined with radiotherapy. Patients with good and poor 5-year prognosis following initial diagnosis were included. The breast cancer data was already normalized so we omitted the preprocessing step. The colon datasets [43] consisted of 62 samples of colon epithelial tissue taken from colon-cancer patients. Sample were obtained both from tumor tissue as well as adjacent, unaffected parts of the colon of the same patients, and measured using high density oligonucleotide arrays. For the analysis of the colon microarray data we followed the same pre-processing approach as we did for the neuromuscular data i.e. we performed log2 transformation and quantile normalization across samples.

Datasets from acute lymphoblastic leukemia (ALL) and acute myeloid leukemia (AML) [6], two distinct acute leukemias, were used for cancer subtype classification. The train set consisted of 27 ALL samples and 11 AML samples. Finally, prostate cancer [44] training data consisted of 52 prostate tumour tissue and 50 normal prostate tissue datasets, while the testing data consisted of 25 tumour and 9 normal datasets [45]. In relation to the preprocessing of the leukemia and the prostate data, we first set the Golub’s floor and ceiling values (floor = 100 and ceiling =16.000), though without filtering the genes, and then applied log10 transformation and quantile normalization across samples. For all cancers datasets we kept the train and test sets as provided, see Table 1.

#### Simulated data

Apart from the real microarray data, we investigated m*AP*-KL’s performance on two synthetic datasets. We intentionally utilized two different simulation setups to examine two different hypotheses. In the first hypothesis, we wanted to verify that m*AP*-KL provides us with a small subset of representative features, at least one gene per cluster, adequate for accurate classification. Therefore, we considered a binary classification problem simulating a normal-disease case with six different scenarios (see Additional files 1, 2, 3, 4, 5, 6, 7, 8, 9) in relation to the number of differentially expressed genes (DEGs) that are included in the disease class samples.

In particular, we started with 50 DEGs belonging to five clusters of 10 ‘genes’ and reached to 500 DEGs spreading in 25 clusters of 20 ‘genes’ per cluster, trying to imitate pathways. The normal and the disease classes have 1,200 samples of 10,000 ‘genes’ per sample, where the first 100 samples from each class compose the train set and the rest form the test set. The non-differentially expressed genes are independently drawn from normal distribution with mean = 0 and variance = 0.5.

In the second hypothesis, we employed a subset of the publicly available ‘Golden Spike’ [46] Affymetrix case–control experiment, incorporated in the ‘st’ package [47,48] under the name ‘choedata’. In this scenario, it was intriguing to explore the number of the known DEGs included in m*AP*-KL’s subset and whether they are capable of providing us with accurate models. The ‘choedata’ describes a binary classification problem with three replicates per class and 1,331 DEGs scattered randomly among 11,475 genes.

### Feature selection methods

We employed 13 feature selection/elimination approaches on the same real microarray datasets and compared its performance with that from m*AP*-KL. We set the subset’s length to 20 top ranked genes for all methods, except for maxT where we additionally engaged the top 200 gene list and evaluate their prediction strength. We decided to include methods that belong to different feature selection categories. In particular, we selected seven univariate filter methods (eBayes, ODP, maxT, SAM, SNR and t-test), one multivariate filter algorithm (cat), three dimension reduction approaches (BGA-COA, PCA, PLS), one embedded method (Random Forest), one hybrid method (HykGene) and one Monte-Carlo like (Rnd) technique.

**Between Group Analysis** (BGA) is a multiple discriminant approach that can be used with any combinations of numbers of genes and samples. BGA uses a conventional ordination technique such as Correspondence Analysis (COA) or principal component analysis (PCA) to carry out ordination of groups of samples. For *N* groups we find *N* − 1 eigenvectors or axes that arrange the groups so as to maximise the between group variances. The individual samples are then plotted along them. Each eigenvector can be used as a discriminator to separate one of the groups from the rest. New samples are then placed on the same axes and can be classified on an axis-by-axis basis or by proximity to the group centroids. It is especially effective when combined with COA because it allows us to examine the correspondences between the grouped samples and those genes which most facilitate the discrimination of these groupings [49].

**The Hybrid system for marker Gene selection** (HykGene) is a hybrid approach that combines sequentially gene ranking and clustering analysis. Firstly, a set of top-ranked informative genes is selected with the aid of filtering algorithms (Relief-F, Information Gain, and x^2^-statistic), and secondly a hierarchical clustering is applied on these genes to generate a dendrogram. Finally, a sweep-line algorithm is used to analyze the dendrogram and marker genes are selected by collapsing dense clusters. The best number of clusters is determined systematically by applying the leave-one-out cross-validation (LOOCV) on the training data, trying all different options for extracting clusters from the dendrogram [28].

**Principal component analysis** (PCA) is a classic and one of the oldest dimension reduction approaches. It searches for linear combinations of the original measurements called principal components (PCs) that can effectively represent effects of the original measurements. PCs are orthogonal to each other and may have dimensionality much lower than that of the original measurements. Because of its computational simplicity and satisfactory statistical properties, PCA has been extensively used in bioinformatics studies, particularly gene expression studies, to reduce the dimensionality of high-throughput measurements and shown to have satisfactory performance [15]. We implement it through the BGA package.

**The optimal discovery procedure** (ODP) is a high dimensional approach that uses all of the relevant information across tests when assessing the significance of each one. It allows us to test multiple hypotheses simultaneously in such a way that the total number of expected true positive results is maximized for each fixed number of expected false positive results. This procedure can be viewed as a multiple-test extension of the Neyman–Pearson (NP) procedure for testing a single hypothesis. This method is available through the EDGE software program [50].

**maxT:** It is a function that computes permutation adjusted p-values for step-down maxT multiple testing procedures as described in Westfall & Young [51], which provides strong control of the family-wise Type I error rate (FWER) [52]. It determines the family-wise error rate adjusted p-values using the Wilcoxon rank sum statistic. To do this the class labels are permuted, and the Wilcoxon statistic for each gene is calculated. The maximum Wilcoxon statistic is recorded for 1,000 random permutations and the p for each gene is estimated as the proportion of the maximum permutation-based t-statistics that are greater than the observed value [22]. This is the ranking approach that we have engaged in m*AP*-KL.

**GenePattern** is a software package, which provides a comprehensive environment that can support (i) a broad community of users at all levels of computational experience and sophistication, (ii) access to a repository of analytic and visualization tools and easy creation of complex analytic methods from them and (iii) the rapid development and dissemination of new methods [53]. The Comparative Marker Selection suite is freely available as a GenePattern module that allow users to apply and compare different methods of computing significance for each marker gene, a viewer to assess the results, and a tool to create derivative datasets and marker lists based on user-defined significance criteria. During our experiment we utilized two test statistics, the t-test and signal-to-noise ratio. From a plethora of estimates related with the significance of each gene we used the “rank” estimate which is based on the value of the test statistic [54].

**t-test**: This is the standardized mean difference between the two classes. It is the difference between the mean expression of class 1 and class 2 divided by the variability of expression, which is the square root of the sum of the standard deviation for each class divided by the number of samples in each class.

**SNR:** The signal-to-noise ratio is computed by dividing the difference of class means by the sum of their standard deviations.

**Partial Least Squares** (PLS) is a highly efficient statistical regression technique that is well suited for the analysis of high-dimensional genomic data. The underlying idea of PLS is to find uncorrelated linear transformations of the original predictor variables which have high covariance with the response variables. These linear transformations can then be used as predictors in classical linear regression models to predict the response variables. Since the p original variables are summarized into a small number of relevant new components, linear regression can be performed even if the number of original variables p is much larger than the number of available observations [55].

**Random Forests** (RF) are a combination of tree-structured predictors where each of the trees grows using a random process. Given a training set with N samples and M features, the N instances are sampled at random (with replacement), so as to generate a random vector Θ for each tree. For the kth tree, there is a random vector Θ_k_ which is independent of the previous random vectors, Θ_1_, … , Θ_k−1_, but with the same distribution for all trees in the forest. Hence, every tree is grown using the training set and its random vector, resulting in a classifier, which votes for the most popular class.

When RF draws the training set for the current tree by sampling with replacement, about one-third of the cases are left out of the sample, and called out-of-bag data (OOB). This OOB data is used to get estimates of variable importance. To measure the importance of variable x_j_, values of x_j_ are permuted in the OOB sample, and the class membership of the OOB samples are predicted again from the tree. The number of correctly classified samples after permutation is subtracted from the original count of correctly classified samples and divided by the number of OOB samples for that tree, thus giving the decrease in classification accuracy as a proportion of samples. This permutation procedure is repeated for each tree in the forest, and the mean decrease in accuracy (MDA) is defined as the average of these values over all trees in the forest (multiplied by 100 and presented as a mean percentage decrease in accuracy). In this experiment, a random forest classifier with 1,000 trees is applied [14].

**Significance Analysis of Microarrays** (SAM) identifies genes with significant changes in gene expression by conducting a set of gene-specific t-tests and then assigning a score to each gene relative to the standard deviation of those tests. Genes are characterized as significant if their score is greater than an adjustable threshold (delta). SAM employs permutations of the repeated measurements in order to estimate the false discovery rate (FDR) i.e. the percentage of genes identified by chance. Through the threshold adjustment, we may conclude to smaller or larger sets of genes [56].

**The empirical Bayes moderated t-statistic** (eBayes) ranks genes by testing whether all pairwise contrasts between different outcome-classes are zero. It is applied to extract information across genes thus making the final analyses more stable even for experiments with limited number of arrays. Moderated t-statistics lead to p-values with increased degrees of freedom for the individual variances hence, reflecting the greater reliability associated with the smoothed standard errors [57]. Linear Models for Microarray Data (Limma) is a package, which incorporates this statistic [58].

**Correlation-adjusted t’-scores** (cat) is the product of the square root of the inverse correlation matrix with a vector of t scores and represents the individual contribution of each single feature (gene) to separate two groups after removing the effect of all other features. This method takes account of correlations among genes before adjusting the t-statistics. In the absence of correlation the cat score reduces to the standard t-score. The cat score offers a simple approach to feature selection, both of individual genes and sets of genes [48].

Apart from these standard methods, we wanted to explore whether the use of a feature selection method over the top 200 list will benefit the prediction or not. Therefore, we decided to select randomly gene probes from the ranked list and then assess its classification performance. In order to control the randomness and finally conclude to a stable outcome we created randomly 10 subsets of 20 gene probes, run the classification process and finally summarized the results. Thus, the **random** (Rnd) scores refer to these mean values per disease.

## Results and discussion

### Overview

Following the development of m*AP*-KL we designed and executed an elaborate set of analytical experiments with 5-CV on the training set and hold-out validation on a separate set to assess its performance across whole genome expression datasets from both small and large patient cohorts. In relation to small cohorts, we employed data from 6 neuromuscular diseases, while for large cohorts we utilized data from 4 different types of cancer. On those microarray datasets, we also applied 13 other feature selection/elimination approaches and compared the classification results (Table 2 and Table 3).

**Table 2:** The FS methods sorted by the AUC metric achieved in validation test for each neuromuscular disease using the RF classifier

	**FS methods**	**5-CV**			**Hold-out Validation**		
		**AUC**	**TNR**	**TPR**	**AUC**	**TNR**	**TPR**
**ALS**	**m*AP*-KL**	1.00 (0.00)	1.00 (0.00)	0.98 (0.14)	**1.00**	1.00	1.00
	**BGA-COA**	1.00 (0.00)	1.00 (0.00)	1.00 (0.00)	**1.00**	1.00	1.00
	**BGA-COA**	1.00 (0.00)	1.00 (0.00)	1.00 (0.00)	**1.00**	1.00	1.00
	**maxT (200)**	1.00 (0.00)	1.00 (0.00)	1.00 (0.00)	**1.00**	1.00	1.00
	**PLS-CV**	1.00 (0.00)	1.00 (0.00)	1.00 (0.00)	**1.00**	0.91	1.00
	**ODP**	1.00 (0.00)	1.00 (0.00)	1.00 (0.00)	**1.00**	0.73	1.00
	**RF-MDA**	1.00 (0.00)	0.98 (0.10)	1.00 (0.00)	**1.00**	0.73	1.00
	**SAM**	1.00 (0.00)	1.00 (0.00)	1.00 (0.00)	**1.00**	0.64	1.00
	**cat**	1.00 (0.00)	1.00 (0.00)	1.00 (0.00)	**1.00**	.64	1.00
	**t-test**	1.00 (0.00)	1.00 (0.00)	1.00 (0.00)	**1.00**	.64	1.00
	**eBayes**	1.00 (0.00)	1.00 (0.00)	1.00 (0.00)	**1.00**	.36	1.00
	HykGene	1.00 (0.00)	0.97 (0.12)	0.98 (0.10)	0.94	0.45	1.00
	maxT	1.00 (0.00)	1.00 (0.00)	1.00 (0.00)	0.94	0.45	1.00
	SNR	1.00 (0.00)	1.00 (0.00)	1.00 (0.00)	0.94	0.73	1.00
	Rnd	1.00 (0.00)	1.00 (0.00)	1.00 (0.00)	0.89	0.70	0.93
	PCA	0.83 (0.30)	0.61 (0.43)	0.77 (0.39)	0.58	0.27	1.00
LGMD2B	**RF-MDA**	1.00 (0.00)	1.00 (0.00)	1.00 (0.00)	**1.00**	0.73	1.00
	**maxT (200)**	1.00 (0.00)	1.00 (0.00)	1.00 (0.00)	**1.00**	0.64	1.00
	**PLS-CV**	1.00 (0.00)	1.00 (0.00)	1.00 (0.00)	**1.00**	0.55	1.00
	BGA-COA	1.00 (0.00)	1.00 (0.00)	1.00 (0.00)	0.98	0.73	1.00
	maxT	1.00 (0.00)	1.00 (0.00)	1.00 (0.00)	0.91	0.64	1.00
	Rnd	1.00 (0.00)	1.00 (0.00)	0.93 (0.25)	0.90	0.56	1.00
	SNR	1.00 (0.00)	1.00 (0.01)	1.00 (0.00)	0.88	0.73	1.00
	HykGene	1.00 (0.00)	1.00 (0.00)	1.00 (0.00)	0.82	0.64	1.00
	t-test	1.00 (0.00)	1.00 (0.00)	1.00 (0.00)	0.82	0.73	0.67
	ODP	1.00 (0.00)	1.00 (0.00)	1.00 (0.00)	0.73	0.45	1.00
	mAP-KL	1.00 (0.00)	1.00 (0.00)	1.00 (0.00)	0.70	0.36	0.67
	SAM	1.00 (0.00)	1.00 (0.00)	1.00 (0.00)	0.52	0.27	1.00
	eBayes	1.00 (0.00)	1.00 (0.00)	1.00 (0.00)	0.48	0.27	0.67
	cat	1.00 (0.00)	1.00 (0.00)	1.00 (0.00)	0.36	0.09	1.00
	PCA	0.89 (0.25)	0.74 (0.38)	0.61 (0.44)	0.21	0.09	1.00
NM	**SNR**	1.00 (0.00)	1.00 (0.00)	1.00 (0.00)	**0.90**	0.77	1.00
	t-test	1.00 (0.00)	0.98 (0.10)	1.00 (0.00)	0.89	0.77	0.80
	HykGene	1.00 (0.00)	1.00 (0.00)	0.99 (0.07)	0.88	0.69	0.80
	maxT (200)	1.00 (0.00)	1.00 (0.00)	1.00 (0.00)	0.80	0.69	0.80
	cat	1.00 (0.00)	1.00 (0.00)	0.99 (0.07)	0.78	0.46	1.00
	mAP-KL	1.00 (0.00)	1.00 (0.00)	1.00 (0.00)	0.74	0.69	0.60
	Rnd	0.98 (0.03)	0.87 (0.09)	0.96 (0.06)	0.67	0.49	0.76
	SAM	1.00 (0.00)	0.87 (0.28)	0.98 (0.10)	0.65	0.15	1.00
	PCA	0.82 (0.30)	0.77 (0.35)	0.73 (0.39)	0.55	0.92	0.40
	BGA-COA	0.96 (0.14)	0.87 (0.28)	0.91 (0.19)	0.47	0.23	0.60
	PLS-CV	0.97 (0.12)	0.87 (0.28)	0.99 (0.07)	0.42	0.08	1.00
	maxT	1.00 (0.00)	1.00 (0.00)	1.00 (0.00)	0.37	0.38	0.40
	ODP	1.00 (0.00)	0.92 (0.23)	1.00 (0.00)	0.25	0.38	0.20
	RF-MDA	1.00 (0.00)	1.00 (0.00)	1.00 (0.00)	0.22	0.15	0.60
	eBayes	-	-	-	-	-	-

**Table 3:** The FS methods sorted by the AUC metric achieved in validation test for each cancer disease using the RF classifier

	**FS methods**	**5-CV**			**Hold-out Validation**		
		**AUC**	**TNR**	**TPR**	**AUC**	**TNR**	**TPR**
**BREAST**	**m*AP*-KL**	0.80 (0.11)	0.79 (0.16)	0.73 (0.18)	**0.87**	1.00	0.50
	maxT(200)	0.85 (0.11)	0.83 (0.13)	0.69 (0.17)	0.83	0.71	0.83
	PLS-CV	0.91 (0.08)	0.85 (0.13)	0.77 (0.15)	0.82	0.86	0.42
	RF-MDA	0.91 (0.07)	0.91 (0.11)	0.70 (0.16)	0.82	0.86	0.75
	maxT	0.87 (0.10)	0.84 (0.13)	0.74 (0.18)	0.77	0.71	0.58
	SAM	0.82 (0.11)	0.79 (0.15)	0.69 (0.19)	0.77	0.71	0.75
	SNR	0.86 (0.10)	0.85 (0.14)	0.72 (0.20)	0.77	0.71	0.67
	BGA-COA	0.83 (0.10)	0.79 (0.15)	0.67 (0.15)	0.76	0.57	0.58
	HykGene	0.91 (0.06)	0.86 (0.12)	0.76 (0.17)	0.76	0.71	0.75
	Rnd	0.79 (0.01)	0.76 (0.03)	0.65 (0.03)	0.76	0.70	0.78
	cat	0.91 (0.07)	0.86 (0.12)	0.78 (0.16)	0.75	0.71	0.50
	PCA	0.72 (0.14)	0.66 (0.18)	0.56 (0.19)	0.75	0.43	0.67
	ODP	0.83 (0.10)	0.80 (0.14)	0.69 (0.18)	0.74	0.71	0.58
	t-test	0.82 (0.10)	0.81 (0.14)	0.69 (0.19)	0.73	0.71	0.58
	eBayes	-	-	-	-	-	-
**COLON**	**m*AP*-KL**	0.99 (0.03)	0.95 (0.12)	0.97 (0.09)	**0.89**	0.71	0.84
	BGA-COA	0.98 (0.06)	0.89 (0.22)	0.87 (0.19)	0.87	0.71	0.80
	Rnd	0.98 (0.02)	0.90 (0.06)	0.90 (0.03)	0.84	0.73	0.82
	maxT(200)	1.00 (0.00)	0.94 (0.13)	0.94 (0.13)	0.83	0.71	0.88
	PCA	0.79 (0.19)	0.80 (0.23)	0.72 (0.26)	0.83	0.43	0.84
	ODP	0.99 (0.03)	0.97 (0.13)	0.93 (0.13)	0.82	0.71	0.80
	HykGene	0.98 (0.06)	0.93 (0.14)	0.95 (0.12)	0.81	0.71	0.88
	RF-MDA	0.99 (0.03)	0.96 (0.11)	0.93 (0.13)	0.81	0.71	0.80
	eBayes	0.99 (0.03)	0.97 (0.11)	0.93 (0.13)	0.80	0.71	0.80
	SAM	1.00 (0.02)	0.99 (0.09)	0.93 (0.13)	0.80	0.71	0.80
	cat	0.99 (0.04)	0.97 (0.14)	0.93 (0.13)	0.80	0.57	0.80
	maxT	1.00 (0.02)	0.97 (0.10)	0.94 (0.13)	0.79	0.71	0.80
	PLS-CV	1.00 (0.02)	0.94 (0.16)	0.94 (0.13)	0.79	0.71	0.80
	SNR	0.99 (0.03)	1.00 (0.00)	0.93 (0.13)	0.79	0.71	0.80
	t-test	0.99 (0.03)	0.99 (0.05)	0.93 (0.13)	0.79	0.71	0.80
**LEUKEMIA**	**BGA-COA**	0.99 (0.04)	1.00 (0.00)	0.81 (0.27)	**1.00**	1.00	0.86
	**maxT(200)**	1.00 (0.00)	1.00 (0.00)	1.00 (0.00)	**1.00**	1.00	0.86
	**eBayes**	1.00 (0.00)	1.00 (0.00)	0.91 (0.19)	**1.00**	0.95	0.93
	RF-MDA	1.00 (0.00)	1.00 (0.00)	1.00 (0.00)	0.99	1.00	0.86
	PLS-CV	1.00 (0.00)	1.00 (0.00)	0.89 (0.25)	0.99	0.95	0.93
	SAM	1.00 (0.00)	1.00 (0.00)	0.91 (0.19)	0.99	0.95	0.93
	cat	1.00 (0.00)	1.00 (0.00)	0.95 (0.14)	0.99	0.95	0.93
	HykGene	1.00 (0.00)	1.00 (0.00)	0.90 (0.20)	0.97	0.90	0.93
	Rnd	0.99 (0.01)	0.98 (0.02)	0.86 (0.06)	0.97	0.99	0.75
	maxT	1.00 (0.02)	0.98 (0.07)	0.85 (0.27)	0.96	1.00	0.64
	m*AP*-KL	1.00 (0.00)	1.00 (0.00)	0.97 (0.17)	0.71	0.90	0.43
	PCA	0.56 (0.16)	1.00 (0.00)	0.00 (0.00)	0.64	0.95	0.14
	SNR	0.50 (0.00)	1.00 (0.00)	0.00 (0.00)	0.50	1.00	0.00
	t-test	0.50 (0.00)	1.00 (0.00)	0.00 (0.00)	0.50	1.00	0.00
	ODP	-	-	-	-	-	-
**PROSTATE**	**SAM**	0.96 (0.04)	0.97 (0.05)	0.88 (0.10)	**0.92**	0.00	1.00
	maxT(200)	0.95 (0.10)	0.95 (0.10)	0.89 (0.10)	0.88	0.00	1.00
	PLS-CV	0.97 (0.03)	0.95 (0.08)	0.92 (0.07)	0.87	0.33	1.00
	eBayes	0.96 (0.04)	0.98 (0.04)	0.89 (0.10)	0.86	0.00	1.00
	RF-MDA	0.97 (0.04)	0.97 (0.06)	0.90 (0.09)	0.83	0.11	1.00
	m*AP*-KL	0.93 (0.06)	0.90 (0.09)	0.85 (0.11)	0.80	1.00	0.36
	BGA-COA	0.95 (0.05)	0.91 (0.09)	0.89 (0.10)	0.73	0.22	0.88
	Rnd	0.93 (0.02)	0.89 (0.04)	0.86 (0.03)	0.70	0.18	0.94
	HykGene	1.00 (0.00)	1.00 (0.00)	1.00 (0.00)	0.69	0.89	0.24
	maxT	0.89 (0.07)	0.88 (0.09)	0.79 (0.13)	0.50	0.00	1.00
	PCA	0.84 (0.09)	0.77 (0.15)	0.75 (0.15)	0.50	0.00	1.00
	SNR	0.50 (0.00)	0.08 (0.27)	0.92 (0.27)	0.50	0.00	1.00
	t-test	0.50 (0.00)	0.08 (0.27)	0.92 (0.27)	0.50	0.00	1.00
	ODP	-	-	-	-	-	-
	cat	-	-	-	-	-	-

We further assessed the m*AP*-KL’s performance towards other feature selection and/or classification studies, conducted on the same cancer datasets. Finally, we engaged two different simulation setups with known structures and investigated m*AP*-KL’s behaviour.

### Neuromuscular diseases data

The use of small cohorts in biomedical research is common in some types of studies such as those of rare diseases. These small cohorts make feature selection algorithms prone to overfitting and thus less reliable [59] compared to larger cohorts. It was therefore intriguing to explore the robustness and generalization of m*AP*-KL on train sets with length ranging from 12 to 20 samples and test sets with 15 to 19 samples respectively (Table 1).

The majority of the methods in ALS and DMD validation achieved the highest classification score (AUC =1.00) except for the HykGene in ALS and the PCA in DMD with AUC scores of 0.64 and 0.61 respectively. Similarly, in 5-CV test, only the BGA-COA and the PCA with AUC scores of 0.98 and 0.48 respectively, deviated from the rule. In JDM although all of the methods achieved the highest AUC score (1.00) during hold-out validation, the respective TNR score was 0.88 for the BGA-COA, eBayes, ODP, SNR and cat methods. In 5-CV the PCA was the only method that failed to distinguish correctly all samples (AUC = 0.90).

In relation to the LGMD2A, ten methods, including the maxT (200), achieved the highest AUC value, though only BGA-COA, m*AP*-KL and maxT (200) achieved the highest TNR and TPR, too. The TNR score for PLS-CV was 0.91, for RF-MDA, ODP and SNR was 0.73, while for HykGene was 0.45 and for eBayes 0.36. It is worth noticing that the TNR score of the maxT with the 20 genes subset, was considerably lower to that of maxT (200). During the 5-CV evaluation, the classification results are almost ideal, since only PCA had an AUC score less than 1.00.

Contrary to the previous datasets, in LGMD2B validation, only three of the methods (RF-MDA, maxT (200) and PLS-CV) achieved the highest AUC (1.00) but their TNR score was 0.73, 0.64 and 0.55 respectively. Although many methods distinguish all disease samples correctly i.e. TPR = 1.00, all of them failed to discern all normal samples i.e. TNR < 1.00. Approximately half of the methods had a TNR below 0.50 (included, eBayes, SAM and m*AP*-KL) and no method had TNR greater than 0.80. On the other hand, the 5-CV classification results were very promising since all methods but PCA achieved the highest score i.e. 1.00 in all three metrics.

Likewise in NM validation, all of the methods faced considerable difficulties in distinguishing disease and normal samples. Only the SNR, the t-test and the HykGene methods managed to reach an AUC score close to 0.90. The TPR results for NM as opposed to those for LGMD2B were discouraging, since only four methods (PLS-CV, cat, SAM, and SNR) classify all the disease samples correctly, and the TPR score for the rest of the methods range from 0.20 to 0.60, with the exception of t-test and HykGene (TPR = 0.80). In contrast, in 5-CV ten methods achieved AUC score of 1.00, though only m*AP*-KL, maxT, maxT (200), RF-MDA, and SNR achieved the optimum score in TNR and TPR metrics. The PLS-CV and BGA-COA had the same TNR score (0.87) but different TPR (0.99 and 0.91) and AUC (0.97 and 0.96). The PCA method achieved the lowest AUC score (0.82) with TNR and TPR scores equal to 0.77 and 0.73. Finally, the eBayes method failed to produce a list of significant genes.

### Cancer data

As far as the large patient cohorts is concerned, we utilized microarray data from four different types of cancer (breast cancer, colon cancer, leukemia, and prostate cancer), with train sets length ranging from 30 to 102 samples and test sets from 19 to 34 (Table 1).

In breast cancer HO validation, m*AP*-KL attained the optimum score (1.00) in TNR metric and the best AUC score (0.87). Two methods, PLS-CV and RF-MDA, achieved competitive TNR and AUC scores of 0.86 and 0.82 respectively. However, all methods faced difficulties to distinguish the non-responsive samples, and except the maxT (200) with a TPR score of 0.83, followed by the Rnd technique (0.78) and the RF-MDA, the HykGene and the SAM methods (0.75). During the 5-CV validation, PLS-CV, RF-MDA, HykGene and cat had an AUC score of 0.91, which was also the highest score attained. The rest of the methods achieved AUC scores between 0.73 and 0.77, but only SAM had a balance performance between TNR and TPR metrics. It is worth noticing that the TPR results for all methods were below the TNR results. The eBayes method failed to fulfil the analysis task.

In relation to colon cancer, m*AP*-KL presented similar classification behaviour to breast cancer, with an AUC score of 0.89 and a more balanced behaviour between TNR and TPR (0.71 and 0.84). Only the BGA-COA method achieved a competitive AUC score of 0.87. The AUC score for the rest of the methods lay between 0.79 and 0.84. Contrary to breast cancer, the TPR scores were higher than the TNR scores and range from 0.80 to 0.84 for all methods. Regarding the TNR metric, all methods but Rnd (0.73) and PCA (0.43) and cat (0.57) achieved the same score of 0.71. The classification results in 5-CV are very promising with AUC values from 0.98 to 1.00 for all of the methods except PCA, which attained an AUC score of 0.79.

Concerning the leukemia datasets, 10 of the methods (BGA-COA, eBayes, RF-MDA, PLS-CV, SAM, cat, HykGene, Rnd, maxT (200) and maxT) performed similarly in both validation tests. Their AUC were close to 1.00 in both cases, and the TNR results were better than the TPR scores. The m*AP*-KL, although achieving high classification scores in 5-CV, failed to predict correctly all AML samples (TPR = 0.43), and as a results its overall performance was 0.71 during the hold-out validation. The PCA, SNR and t-test methods failed to predict any of the 14 AML samples, although they identified all or almost all of the ALL samples. The ODP algorithm failed to analyse the colon dataset.

Finally, in prostate cancer, no method succeeded in discriminating the samples in both types of validation, alike to NM in neuromuscular diseases section. Even more importantly, during the hold-out validation, many of the methods (eBayes, SAM, maxT (200), maxT, PCA, SNR and t-test) failed to identify even a single sample from the normal class. Only the HykGene excelled in this metric with a TNR score equal to 0.89. However, because of the normal: disease ratio (9 normal and 25 disease samples), the AUC values of eBayes (0.86) and SAM (0.92) are a little deceptive. On the other hand, PLS-CV and m*AP*-KL appear to have an opposite behaviour in relation to TNR and TPR metrics, but the normal: disease ratio tips the scales in favour of PLS-CV (AUC = 0.87). Two algorithms, ODP and cat, could not deal with the prostate data.

### Analysis of previous experiments

At a different level of assessment, we compared the m*AP*-KL’s classification results of the specific cancer datasets, against those published in previous classification studies of the same data. For the purposes of this comparison, we have cited the author’s name, the classification type, the number of the features used, and finally the achieved accuracy (ACC). Since we utilized three different classifiers to build and test m*AP*-KL’s models, in this comparison we present all three results achieved.

In relation to the van ’t Veer et al. [23] breast cancer datasets, we present the classification results from 9 different approaches stemming from 7 studies, see Table 4. Regarding the CV test, Hassan et.al [21] and Hu et al. [60] achieved ACC above 90.00%, higher than van ’t Veer et al. and with less features. However, they utilized all of the samples contrary to van ’t Veer et al. Our method achieved moderate results (ACC = 75.93%) as absolute numbers for the 78 samples but with only 6 features and 5-CV contrary to LOO-CV that engaged by the others. In the hold-out test, although the ACC of m*AP*-KL is the lowest score, we did manage to identify correctly all responsive samples. However, we should consider why we discern only half of the non-responsive samples (type II error).

**Table 4:** An overview of the published classification results in van ’t Veer et al. breast cancer data

**Authors**	**Cross Validation**		**Train-Test**		**Features**
	**Samples**	**Accuracy (%)**	**Samples**	**Accuracy (%)**	
[23]	65/78	83.3	17/19	89.5	70
[21]	-	92.13	-	91.67	3
[61]	60/78	76.90	15/19	78.9	231
[61]	-	76.20	15/19	78.9	231
[61]	62/78	81.40	17/19	89.5	44
[60]	88/97	90.7	-	-	50
[62]	49/78	62.9	-	-	-
[63]	-	-	17/19	89.47	834
[38]	66/97	68.04	-	-	8
m*AP*-KL (RF)	-	75.93	13/19	68.42	6
m*AP*-KL (KNN)	-	56.35	5/19	26.32	6
m*AP*-KL (SVM-linear)	-	71.47	11/19	57.89	6

Fourteen methods employed the Alon et al. [43] colon cancer datasets to assess their classification performance, see Table 5. During the CV assessment we achieved ACC = 96.00% with RF and KNN classifiers higher than the one achieved by Tan and Gilbert [63] (95.16%). Regarding the hold-out validation, Li et al. [64], Nguyen and Rocke [65] and Furey et al. [66] achieved ACC of 94.1%, 93.5% and 90.30% respectively. We reached to 81.25% and 87.50% ACC with 20 genes contrary to Nguyen and Rocke with 50 genes.

**Table 5:** An overview of the published classification results in Alon et al. colon cancer data

**Authors**	**Cross Validation**		**Train-Test**		**Features**
	**Samples**	**Accuracy (%)**	**Samples**	**Accuracy (%)**	
[67]	57/62	91.94	-		-
[67]	53/62	85.48	-		-
[66]	-	-	-	90.3	-
[64]	-	-	-	94.1~	-
[68]	-	-	-	80.6	-
[68]	-	-	-	74.2	-
[68]	-	-	-	72.6	-
[65]	-	-	-	87.1	-
[65]	-	-	-	87.1	-
[65]	-	-	-	93.5	50
[65]	-	-	-	91.9	1000
[69]	52/62 (MAVE-LD)	83.87	-	-	50
[60]	56/62	90.3	-	-	50
[63]	59/62	95.16	-	-	135
m*AP*-KL (RF)	-	96.00	26/32	81.25	20
m*AP*-KL (KNN)	-	96.00	26/32	81.25	20
m*AP*-KL (SVM-linear)	-	94.00	28/32	87.50	20

The ALL/AML discrimination in the leukemia datasets, Table 6, as first presented by Golub et al. [6], is the one most often analyzed among the datasets considered. More than 16 studies and 29 methods have based their evaluation on this set of data. Comparing m*AP*-KL to Golub classification results, we notice that in CV we identify one more sample, whereas in hold-out we misclassify two samples from Golub, though we did that with only 5 genes. There are many methods that distinguish correctly all samples in CV although only Hewett and Kijsanayothin [38] achieved an ACC of 98.61% with only two genes, but using all of the 72 samples. Regarding the hold-out validation, several methods achieved high classification scores with ACC above 95.00%, though only Mukherjee et al. [70] reached the 100%, with only 40 genes. Liu et al. [67] predict correctly all samples in both validation assessments, but we are unaware of the subset’s length. Finally, Singh et al. [40] first employed the specific prostate cancer datasets and we have included the results from three studies, Table 7. m*AP*-KL with the aid of SVM-linear classifier, misclassified one sample in hold-out validation just like Liu et al. [67]. However, in CV we misclassified approximately eight samples more than Liu et al., but with only 12 genes.

**Table 6:** An overview of the published classification results in Golub et al. ALL/AML leukemia data

**Authors**	**Cross Validation**		**Train-Test**		**Features**
	**Samples**	**Accuracy (%)**	**Samples**	**Accuracy (%)**	
[6]	36/38	94.73	29/34	85.29	50
[67]	38/38	100.00	34/34	100	-
[67]	-	-	33/34	97.06	-
[64]	-	-	-	94.1	-
[66]	-	-	-	94.1	-
[68]	-	-	-	91.6	-
[68]	-	-	-	94.4	-
[68]	-	-	-	95.8	-
[65]	-	-	-	94.17	50
[65]	-	-	-	95.44	50
[65]	-	-	-	95.94	50
[65]	-	-	-	96.44	50
[70]	38/38	100	31/34	91.17	7129
[70]	38/38	100	34/34	100	999
[70]	38/38	100	32/34	94.11	99
[70]	38/38	100	30/34	88.23	49
[70]	-	-	34/34	100	40
[70]	-	-	32/34	94.11	5
[71]	-	-	-	95.0~	-
[71]	-	-	-	95.0~	-
[71]	-	-	-	95.0~	-
[72]	37/38	98	34/34	100	185
[73]	38/38	100	34/34	100	3800
[74]	37/38	98	32/34	94.11	21
[62]	71/72	98.6	-	-	-
[38]	71/72	98.61	-	-	2
[69]	38/38 (DLDA)	100	33/34 (DLDA)	97.06	50
[60]	38/38	100	-	-	50
[63]	-	-	31/34	91.18	1038
m*AP*-KL (RF)	-	98.93	24/34	70.59	5
m*AP*-KL (KNN)	-	93.61	24/34	70.59	5
m*AP*-KL (SVM-linear)	-	97.36	27/34	79.41	5

**Table 7:** An overview of the published classification results in Singh et al. prostate cancer data

**Authors**	**Cross Validation**		**Train-Test**		**Features**
	**Samples**	**Accuracy (%)**	**Samples**	**Accuracy (%)**	
[67]	98/102	96.08	33/34	97.06	-
[63]	-	-	25/34	73.53	3071
[38]	124/136	91.18	-	-	6
m*AP*-KL (RF)	-	87.33	18/34	52.94	12
m*AP*-KL (KNN)	-	82.22	29/34	85.29	12
m*AP*-KL (SVM-linear)	-	87.82	33/34	97.06	12

### Biological relevance of discriminatory gene lists

The power of the proposed FS approach is evident not only from its performance in the statistical metrics, but also from the biological relevance of the selected genes either to a broad range of different molecular pathways and biological processes or more importantly to the respective pathological phenotypes. Representative examples include the genes COL3A1, SPARC and PTTG1IP, which are related to extracellular matrix formation and fibroblast growth, biological processes consistent with the increased fibrosis that is observed in skeletal muscles affected by DMD [75]. In ALS the selected genes FHL2 and ALDOA have been directly implicated in muscle function and pathology [76,77] while the multiple genes implicated in the translational process support previous reports on an ALS mouse model [78]. In NM and LGMD2B, the structure associated MYH3, MYH7 and PFN2 genes were depicted, in agreement with the reports of cytoskeletal disorganization in the affected muscle fibers of these patients [41,79]. As opposed to the other skeletal muscle diseases included in this study, JDM is an inflammatory myopathy of presumed autoimmune dysfunction. Consistently with the disease pathology, multiple short-listed genes (CCL5, PCGF2, IFITM1, ISG20) are related to interferon or to chemokine and cytokine production, all key molecules of the immune system [80].

These findings jointly, demonstrate that despite their small size, the discriminatory ‘lists of selected genes’ (see Additional file 4) depicted by the proposed FS approach contain biologically relevant genes, representative of the respective disease related molecular pathways.

### Simulation studies

#### i. The clusters setup

We applied the m*AP*-KL on training sets of 200 samples with 10.000 ‘genes’ and diverse number of DEGs. Moreover, for each training set we differentiated the number of the top ranked genes kept for clustering (see Table 8). The purpose of this case study was twofold. On the one hand, we wanted to investigate how many DEGs are included in our final subset along with their cluster origin. Furthermore, we explored the influence on the DEGs’ selection when varying the number of top ranked genes. We also employed three other FS methods, (eBayes, maxT and RF-MDA), keeping either the top 20 ranked ‘genes’ (cases of 50 DEGs, 100 DEGs, 200 DEGs, 300 DEGs) or the top 30 ranked ‘genes’ (cases of 400 DEGs and 500 DEGs) trying to keep their length comparable with the subset’s length of m*AP*-KL.

**Table 8:** **The number of clusters identified by m*AP*-KL for several top *N *ranked genes compared to three other FS methods (the number of genes per subset is in parenthesis)**

				**Identified Clusters**									
**DEGs**				**Top ranked genes (m*AP*-KL)**							**eBayes**	**maxT**	**RF-MDA**
	**50**	**100**	**150**	**200**	**250**	**300**	**350**	**400**	**450**	**500**			
50	5 (5)	6 (6)	4 (4)	3 (3)	3 (3)	3 (3)	2 (2)	2 (2)	2 (2)	2 (2)	2 (20)	2 (20)	5 (20)
100	3 (3)	5 (5)	6 (6)	6 (14)	5 (5)	4 (4)	4 (4)	4 (4)	3 (3)	3 (3)	1 (20)	2 (20)	5 (20)
200	3 (3)	6 (6)	8 (8)	10 (10)	11 (11)	11 (11)	8 (8)	5 (5)	5 (5)	5 (5)	1 (20)	2 (20)	10 (20)
300	3 (3)	6 (6)	8 (8)	10 (10)	13 (13)	15 (15)	11 (11)	7 (7)	7 (7)	6 (6)	2 (20)	4 (20)	10 (20)
400	4 (4)	6 (6)	8 (8)	11 (11)	13 (13)	15 (15)	18 (18)	20 (20)	21 (23)	10 (10)	3 (30)	4 (30)	16 (30)
500	4 (4)	7 (7)	9 (9)	11 (11)	13 (13)	16 (16)	18 (18)	20 (20)	23 (23)	25 (25)	3 (30)	4 (30)	19 (30)

As far as the identification of DEGs belonging to different clusters is concerned, the m*AP*-KL managed to compose subsets with at least one representative ‘gene’ from each cluster. Besides, as shown in Table 8, in almost all cases the maximum subsets’ length does not exceed the actual number of clusters in the training set. In relation to the other FS methods, only the RF-MDA method composed subsets of ‘genes’ with satisfactory representation of the actual clusters and comparable to mAP-KL. The eBayes and maxT methods demonstrated poor enrichment.

With respect to the effect of the number of top ranked ‘genes’ kept for clustering, it is evident that the closer to the real number of DEGs, the better the identification and selection of representative genes. Specifically, in cases where the number of DEGs is considerably lower than the number of *N* top ranked genes (e.g. 50 DEGs with 200 top ranked genes) the identified clusters are less than the actual. Similarly, when the number of DEGs far exceeds the number of *N* top ranked genes the identified clusters are fewer, for instance 500 DEGs with 200 top ranked genes parameter. Nonetheless, during the real gene expression data experiment, we employed a moderate value for the parameter N = 200 top ranked genes.

As a final point, we formed the respective train-test sets for all methods and evaluated their performance with the aid of three classifiers (SVM-linear, KNN, RF). All methods performed accurately (ACC = 100%) for all three classifiers, see Additional file 7.

#### ii. The ‘choedata’ setup

In this setup, we were interested in exploring, the length of the m*AP*-KL’s subset in relation to the known DEGs included in it. Therefore, we applied on the ‘choedata’ the m*AP*-KL, engaging a non-parametric and a parametric statistical methods Table 9. We observed that the parametric Welch-t test, led us to a subset of 16 genes with 13 DEGs included, whereas the non-parametric Wilcoxon’s test, concluded to a subset of 15 genes with only 8 DEGs.

**Table 9:** **The subsets of genes selected from the ‘choedata’ according to m*AP*-KL**

**Wilcoxon**		**Welch-t**	
**Symbol**	**Position**	**Symbol**	**Position**
**tun**	17	**Rim**	7983
**CG6904**	21	CG14254	8561
**SH3PX1**	53	**Cyp4p2**	9874
**CG10283**	66	**CG10483**	10011
**Tgt**	92	**CG8193**	593
**CG17930**	114	**Gdh**	11006
CG8300	120	**CG17600**	3545
**b**	123	**Gprk2**	11303
**CG12213**	162	kek3	2322
RhoGEF2	163	**CG5880**	10244
Imp	188	**CG3544**	9612
Dip2	209	**CG4785**	11063
Spred	219	**CG32043**	1148
NA	269	CG18125	2424
NA	333	**CG7069**	9585
		**orb**	9432

We then formed classification models with the assistance of three classifiers (SVM-linear, KNN, RF) and assessed their performance. Despite this remarkable difference in the number of DEGs included in the two subsets, the classification results were accurate in both cases. Nonetheless, including more DEGs in a classifier is of benefit to the biological analysis if not to the classification process itself.

## Conclusions

**Figure 2:**
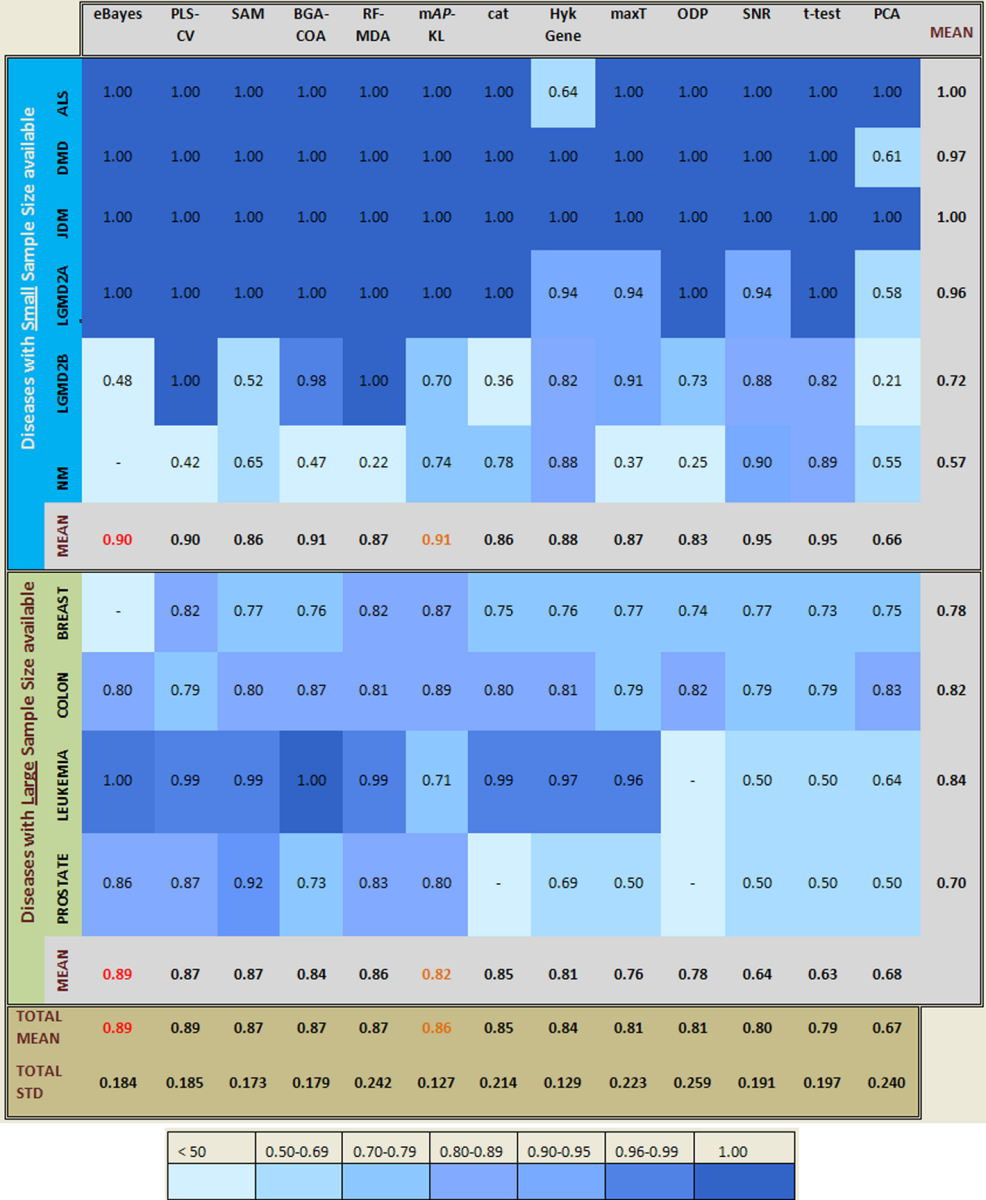
**The overall performance of the FS methods according to the AUC metric.** We have sorted the methods, except the Rnd, which is not actually a method, according to the mean of the AUC values. The standard deviation across all diseases quantifies the robustness of each method. The mean value per disease across all feature selection methods is a difficulty index of discrimination. The NM from the myopathies and the prostate cancer were the most difficult cases towards the phenotype discrimination.

The proposed hybrid FS method (m*AP*-KL), demonstrates how effective the combination of a multiple hypothesis testing approach with a clustering algorithm can be to select small yet informative subsets of genes in binary classification problems. Across a variety of diseases and number of samples, m*AP*-KL presents competitive classification results (Figure 2), compared to other FS methods and specifically to the HykGene method, which follows a similar philosophy, first ranking and then clustering. However, we discern an unbalanced behaviour between the TNR and TPR metrics. In particular, the m*AP*-KL outperforms the other FS methods regarding the control of the type I error but underperforms with regard to the type II error. This issue is under ongoing investigation so as to further improve the efficiency of our method.

Apart from the classification performance, its data-driven and classifier independent features characterize m*AP*-KL. Indeed, the engagement of a cluster quality index diminishes any fuzziness and provides the clustering algorithm with a representative number of potential clusters, as clearly presented in the first simulation data setup. Hence, the data determine the size of the subset and the clustering algorithm decides on which informative genes are to be included. Since no classifier takes part during the subset construction, our subsets perform efficiently across several classification algorithms, for instance SVM-linear, KNN and RF. A further advantage of the employment of m*AP*-KL is that the clustering correlation on the gene expression values may reflect biological relevance of the selected genes with the respective disease, thus providing a reasonable basis for discovering prognostic biomarkers [81].

Finally, we would like to highlight some points of interest in relation to ranked gene lists, which retrospectively confirm our initial motivation towards the m*A*P-KL’s implementation. In particular, a subset of 200 or more top ranked genes, may lead to accurate classification as demonstrated by the results of maxT (200), which achieved outstanding classification results with AUC = 0.97 in neuromuscular diseases and AUC = 0.89 in cancers, but such a lengthy subset may contain a number of irrelevant genes that will act as “noise” when performing further biological analysis. On the other hand, keeping a subset of top *N* genes, where N = 5,10,…n, needs several rounds of “trial and error” attempts before concluding to the best *N* value. Otherwise setting the *N* parameter arbitrarily does not guarantee robust and efficient classification results, as shown in the case of the 20 genes subset of the maxT. Additionally, forming subsets by selecting genes randomly from an already ranked list may lead to satisfactory classification results. The Rnd technique achieved comparable classification results to maxT either with 200 or 20 genes subset. However, the subsets are not reproducible and no biological evidence can be inferred for them. Taking into account all the aforementioned issues, we claim that the novelty and strength of m*AP*-KL is the efficient sampling of the ranked gene list, selecting those genes that are necessary for improved classification, rather than keeping just a predefined number of top N ranked genes.

## Competing interests

The authors declare that they have no competing interests.

## Authors’ contributions

AS, DS and GS conceived this project. AS carried out all the experiments and the analysis as principal investigator. DS carried out the biological relevance analysis and wrote the relevant section. AS wrote the rest of the paper. GS supervised this project. All authors read and approved the final manuscript.

## Supplementary Material

Additional file 1 **This function implements in R-code, the m*AP*-KL’s functionality.** Click here for file

Additional file 2 In this file, we present the 5-CV classification results for all real microarray data, when using three different classifiers (SVM-linear, KNN, and RF). Click here for file

Additional file 3 In this file, we present the Hold-out validation results for all real microarray data, when using three different classifiers (SVM-linear, KNN, and RF). Click here for file

Additional file 4 **In this file, we have cited the subsets of genes according to the m*AP*-KL method.** Click here for file

Additional file 5 **Contains the microarray data used in this experiment.** For each disease, we provide the ‘class_labels.csv’, ‘train.csv’ and ‘test.csv’ files, which represent the analogy of samples as described in table 1. The intensity values are unprocessed. Click here for file

Additional file 6 In this file, we have cited the clustering setup parameters, the DEGs position per simulation dataset, as well as the DEGs identified per method. Click here for file

Additional file 7 **In this file, we present the classification results of (m*AP*-KL, eBayes, maxT, RF-MDA) in the first simulation setup, where the clustering identification was under investigation.** We employed three classifiers (SVM-linear, KNN, and RF). Click here for file

Additional file 8 **In this file, we present the classification results in the ‘choedata’ when using two different m*AP*-KL’s subsets, stemming from two different ranking approaches.** We used the SVM-linear, KNN, and RF classifiers to assess their performance. Click here for file

Additional file 9 **This file contains the relevant scripts and functions for generating the simulated data.** The ‘clusterSim’ r-package is required. Click here for file
